# Complement-Opsonized Nano-Carriers Are Bound by Dendritic Cells (DC) via Complement Receptor (CR)3, and by B Cell Subpopulations via CR-1/2, and Affect the Activation of DC and B-1 Cells

**DOI:** 10.3390/ijms22062869

**Published:** 2021-03-11

**Authors:** Monika Bednarczyk, Carolina Medina-Montano, Frederic Julien Fittler, Henner Stege, Meike Roskamp, Michael Kuske, Christian Langer, Marco Vahldieck, Evelyn Montermann, Ingrid Tubbe, Nadine Röhrig, Andrzej Dzionek, Stephan Grabbe, Matthias Bros

**Affiliations:** 1 Department of Dermatology, University Medical Center Mainz, Langenbeckstraße 1, 55131 Mainz, Germany; m.bednarczyk.09@aberdeen.ac.uk (M.B.); gmedinam@students.uni-mainz.de (C.M.-M.); ffittler@students.uni-mainz.de (F.J.F.); Henner.Stege@unimedizin-mainz.de (H.S.); mikuske@uni-mainz.de (M.K.); monterma@uni-mainz.de (E.M.); tubbe@uni-mainz.de (I.T.); n.roehrig@uni-mainz.de (N.R.); stephan.grabbe@unimedizin-mainz.de (S.G.); 2 Miltenyi Biotec GmbH, Friedrich-Ebert-Strasse 68, 51429 Bergisch Gladbach, Germany; meiker@miltenyi.com (M.R.); christianlan@miltenyi.com (C.L.); MarcoV@miltenyi.com (M.V.); Andreasd@miltenyibiotec.de (A.D.)

**Keywords:** nanocarrier, carbohydrate surface, complement activation, complement receptor 3, complement receptor 4, dendritic cell, B-1, B-2

## Abstract

The development of nanocarriers (NC) for biomedical applications has gained large interest due to their potential to co-deliver drugs in a cell-type-targeting manner. However, depending on their surface characteristics, NC accumulate serum factors, termed protein corona, which may affect their cellular binding. We have previously shown that NC coated with carbohydrates to enable biocompatibility triggered the lectin-dependent complement pathway, resulting in enhanced binding to B cells via complement receptor (CR)1/2. Here we show that such NC also engaged all types of splenic leukocytes known to express CR3 at a high rate when NC were pre-incubated with native mouse serum resulting in complement opsonization. By focusing on dendritic cells (DC) as an important antigen-presenting cell type, we show that CR3 was essential for binding/uptake of complement-opsonized NC, whereas CR4, which in mouse is specifically expressed by DC, played no role. Further, a minor B cell subpopulation (B-1), which is important for first-line pathogen responses, and co-expressed CR1/2 and CR3, in general, engaged NC to a much higher extent than normal B cells. Here, we identified CR-1/2 as necessary for binding of complement-opsonized NC, whereas CR3 was dispensable. Interestingly, the binding of complement-opsonized NC to both DC and B-1 cells affected the expression of activation markers. Our findings may have important implications for the design of nano-vaccines against infectious diseases, which codeliver pathogen-specific protein antigen and adjuvant, aimed to induce a broad adaptive cellular and humoral immune response by inducing cytotoxic T lymphocytes that kill infected cells and pathogen-neutralizing antibodies, respectively. Decoration of nano-vaccines either with carbohydrates to trigger complement activation in vivo or with active complement may result in concomitant targeting of DC and B cells and thereby may strongly enhance the extent of dual cellular/humoral immune responses.

## 1. Introduction

In the field of biomedical applications, nanocarriers (NC) have gained increasing interest based on their capacity to codeliver different drugs and biologicals in a targeting manner to distinct cell types [1]. Targeted delivery may reduce the overall amount of payload required for therapeutic applications as well as adverse effects caused by effects of drugs on non-target cells. However, NC most often accumulate serum factors on their surface, termed protein corona [2]. On one hand, the protein corona may shield targeting moieties and thereby attenuate intended target cell binding. On the other hand, NC-adsorbed serum factors may engage receptors expressed by non-target cells resulting in unwanted uptake. In this regard, Zhang and coworkers first identified constituents of the protein corona of gold nanoparticles [3]. Then, these NC were incubated with serum deficient of single constituents to elucidate their individual role for the overall composition of the protein corona and corona-dependent NC binding to macrophages. By this approach, antithrombin III, fibronectin and other factors of the protein corona were shown to bind IgG. This, in turn, resulted in elevated binding of the antibody-opsonized NC to Fc receptors on macrophages. The cellular interaction of NC may also be affected in case NC surface properties affect the conformational state of adsorbed proteins. For example, Mortimer and coworkers demonstrated that albumin that adsorbed onto silicate-based NC altered its conformation, which resulted in its recognition by class A scavenger receptors (SR-A) on macrophages. To limit the unwanted binding of NC, especially to myeloid cells, NC have been decorated with polyethyleneglycol (PEG) and dysopsonic proteins, which serve to minimize the formation of a protein corona [2,4] [5,6]. Moreover, We [7] and others [8] have demonstrated that solid core NC coated with the carbohydrate dextran (DEX) to ensure biocompatibility may trigger the carbohydrate complement pathway in vivo resulting in the deposition of active complement C3 fragments. C3-opsonized DEX-coated NC were reported to engage blood neutrophils (polymorphonuclear granulocytes; PMN) via engagement of the complement receptor (CR)3, a heterodimer composed of CD11b and CD18 [9]. However, in other studies, DEX-coated NC were demonstrated to bind SR-A, but not CR3 [10,11,12,13]. We showed that in the spleen, complement-opsonized NC preferably targeted splenic B cells [7] by engagement of CR1/2 [14].

CR3 is commonly expressed by myeloid cells, comprising monocytes/macrophages (MAC), PMN and conventional dendritic cells type 2 (cDC2), enabling efficient binding and internalization of complement-opsonized pathogens as well as of immune complexes [9]. Interestingly, CR3 is also apparent on natural killer (NK) cells [15] and a minor B cell subpopulation, termed B-1 [16], known to contribute to pathogen clearance by phagocytosis, production of IgM, and to confer T cell activation [17].

CR3 also binds numerous serum factors [9], has also been implicated in Toll-like receptor (TLR)/MyD88 signaling processes, and triggering of CR3 was shown to result in inhibitory cell signaling [18,19]. In mice, CR4 (CD11c/CD18) [20] constitutes a pan-DC marker [21], and therefore, is frequently used to identify this important population of antigen-presenting cells (APC) [22]. In contrast to CR3, CR4 has not been reported to display broad substrate affinities and signaling effects when triggered.

Given the important role of DC for the initiation and shaping of immune responses, we wanted to delineate the relative importance of CR3, as expressed by cDC2 only, and of CR4 as expressed by all (murine) DC, for the binding/uptake of complement-opsonized NC. Furthermore, due to the general finding of strong accumulation of most types of NC in the liver, including DEX-coated NC [7,23], we asked for the role of complement factors in this regard.

We show here that within liver DC, MAC and liver sinusoidal endothelial cells (LSEC) bound complement-opsonized NC to an increased extent. However, similar results were obtained using serum devoid of complement activity, suggesting the involvement of other receptors. SR-A, which have been frequently discussed in this regard [10,12,13], did not contribute to NC engagement. In the spleen, all CR1/2- and CR3-expressing types of leukocytes engaged NC at a much stronger extent only when these were complement-opsonized. In general, we observed no functional role of SR-A. Furthermore, by using both CD11b^−/−^ DC as well as CD11b-blocking antibodies, we show that CR3, but not CR4 was critical for binding/uptake of complement-opsonized NC. Finally, within B cells, we identified the B-1 subpopulation as highly active in NC binding, with CR-1/2 playing a major role. In the case of DC and B-1 cells, NC engagement attenuated the expression of activation markers after subsequent LPS-mediated stimulation. Our results suggest that nano-vaccines, which codeliver antigen and native protein adjuvant and address DC and B cells in a complement-dependent manner, may be capable of inducing profound cellular and humoral immune responses.

## 2. Results

To delineate the role of activated complement as a constituent of the protein corona of an NC for its binding to immune cells, we comparatively assessed the binding characteristics of fluorescence-labeled NC coated with the carbohydrates dextran (DEX), solid iron oxide core (FeO-DEX) and bionized nanoferrite (BNF-DEX) and starch (BNF-starch), and included albumin-based NC as an internal control (Table S1).

### 2.1. B Cells and CR3-Expressing Immune Cell Types in Spleen Bind Complement-Opsonized NC at High Extent

**Figure 1:**
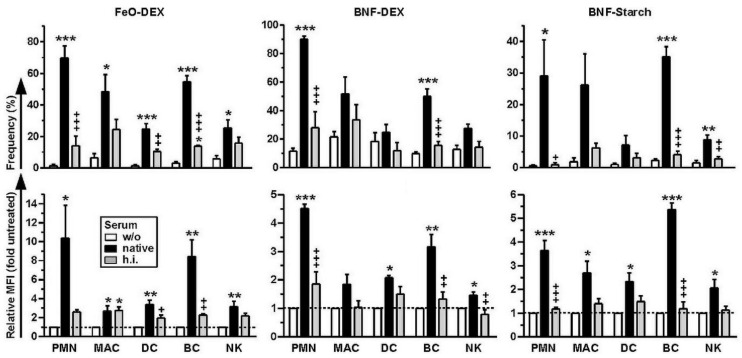
Carbohydrate-coated nanocarriers (NC), when pre-incubated with native serum, engage complement receptor-expressing splenic cell populations at a high extent. Spleen cells were incubated for 3 h with differentially pretreated (left untreated (w/o), pretreated with native or heat-inactivated (h.i.) serum) types of carbohydrate-coated NC. NC binding to neutrophils (PMN), macrophages (MAC), DC, B cells (BC) and natural killer cells (NK) was assessed by FACS analysis. Details on the gating strategy are given in Figure S1. Graphs denote the frequencies of NC-positive cells (upper panel) and NC-dependent MFI (lower panel) of various splenic leukocyte populations (mean ± SEM of 3–4 experiments). Statistical differences versus *w/o serum and ^+^native serum are indicated (one-way ANOVA, Tukey’s test). *^,+^ *p* < 0.05, **^,++^ *p* < 0.01, ***^,+++^ *p* < 0.001.

All myeloid cell types, comprising neutrophils (PMN, polymorphonuclear granulocytes), macrophages, and (conventional) DC, as well as NK cells and B cells, engaged carbohydrate-coated NC to a higher extent when these were pretreated with native mouse serum to enable adsorption of active complement on the NC surface (Figure 1). This effect was largely observed both with regard to the frequencies of NC-binding cells (Figure 1, upper panel) and the mean fluorescence intensities (MFI) of cell-bound NC (Figure 1, lower panel). This effect was largely abrogated when heat-inactivated (h.i.) serum, devoid of complement activity, was used for NC pretreatment. Blocking studies using dextran sulfate as a well-established inhibitor of SR-A, and the corresponding control agent chondroitin sulfate, ruled out the involvement of SR-A (Figure S2).

In the case of albumin NC, only B cells displayed considerably higher binding in the case of pretreatment with native serum (Figure S3). All splenic immune cell types assessed showed attenuated binding of albumin NC pretreated with hi. Serum as compared to non-treated NC. These observations rule out the involvement of CR for the binding of albumin NC. Altogether, our observations indicate that specifically carbohydrate-coated NC, when pre-incubated with native serum, engage complement receptor-expressing splenic cell populations to a higher extent as compared to NC applied w/o pretreatment or pre-incubated with h.i. serum. These findings suggest an important role of complement opsonization of NC for subsequent binding by splenic leukocytes.

For numerous types of NC, considerable accumulation in the liver has been noted [24]. Besides Kupffer cells, which constitute the major liver-resident MAC population, also DC and LSEC, which exert immune functions as well, were reported to internalize NC [24]. To delineate the potential of these cell types to bind NC in a complement-dependent manner, liver NPC (nonparenchymal cells) that comprise the aforementioned liver cell types were isolated and incubated with differentially pretreated NC. Then, engagement of NP liver macrophages, DC and LSEC was assessed (Figure S4).

As shown in Figure S5A, LSEC and DC displayed stronger binding of BNF-DEX and BNF-Starch pre-incubated with native as well as h.i. mouse serum. These findings suggest that other types of receptors than CR may be involved in NC binding. Blocking studies suggested that SR-A did not contribute to the binding of differentially pretreated FeO-DEX (Figure S5B). The protein corona formed around albumin NC by incubation with native or h.i. serum had no effect on binding by liver NPC. Taken together, these observations suggest that MAC and DC in the liver bind complement-opsonized NC by additional/other receptors than the corresponding cell type in the spleen (see Figure 1).

DC constitute the most potent type of APC, and DC in secondary lymphoid organs are frequently addressed in NC-based vaccination approaches. Our finding of complement-mediated binding of carbohydrate-coated NC to this cell type prompted us to perform an in-depth analysis of receptors involved in NC engagement by this cell type. In mice, DC are identified as CD11c^+^ cells, which thereby express CR4 (CD11c/CD18). Splenic DC largely comprise CD11b-positive cDC2, which co-express CR3 and CR4, and CD11b-negative DC that constitute either cDC1 or plasmacytoid (p) DC. As exemplified for FeO-DEX, cDC2 engaged this type of NC to a higher extent than cDC1/pDC at either condition (Figure S6). This finding may indicate DC subpopulation-specific differences in the capacity to engage NC, but may also suggest a role of CR3 in this regard.

### 2.2. Concomitant Expression of CR4 Does Not Increase Binding of Serum-Pretreated NC to DC

To delineate specifically the relative contribution of CR3 and CR4 for the binding and uptake of complement-opsonized NC by DC, we set up cultures in which bone marrow (BM) progenitor cells were differentiated using GM-CSF [25]. After one week, differentiated cells commonly express CR3 (CD11b/CD18) (Figure S7, upper panel), and a high-frequency co-expresses CR4 (CD11c/CD18), termed (inflammatory) BMDC (CD11b^+^CD11c^+^). The minor fraction of CD11b^+^CD11c^−^ (CR3^+^) cells is considered as MAC.

**Figure 2:**
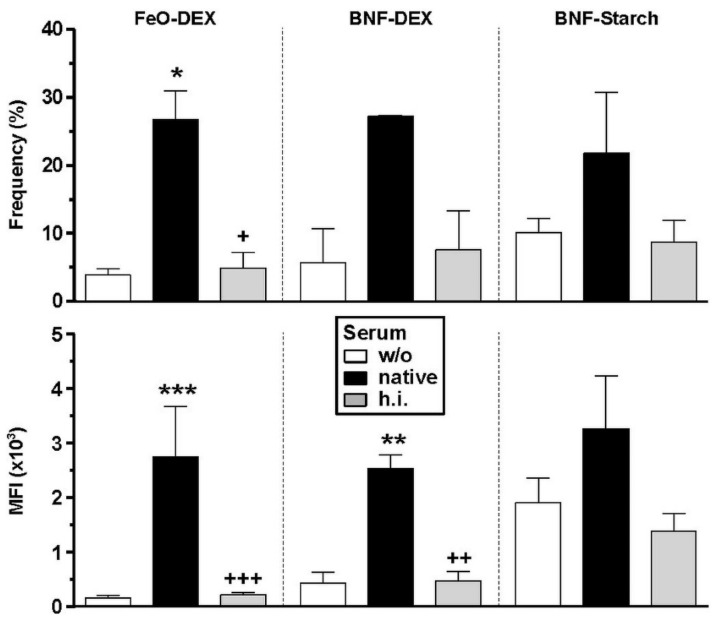
BMDC that express CR3 and CR4 engage carbohydrate-coated NC to a higher extent when pre-incubated with native serum. BMDC were incubated for 3 h with differentially pretreated (left untreated (w/o), pretreated with native or heat-inactivated (h.i.) serum) carbohydrate-coated types of NC. NC binding to CD11c^+^ DC was assessed by flow cytometry and is given as MFI (mean ± SEM of 3 experiments). Details on the gating strategy are given in Figure S7. Statistical differences versus * w/o serum and ^+^native serum are indicated (one-way ANOVA, Tukey’s test). *^,+^ *p* < 0.05, **^,++^ *p* < 0.01, ***^,+++^ *p* < 0.001.

As observed for splenic cells, CD11c^+^ BMDC, which thereby co-express CR3 and CR4, bind all types of carbohydrate-coated NC to a higher extent when these were complement-opsonized due to pre-incubation with native mouse serum as compared to NC left untreated or pre-incubated with h.i. serum (Figure 2). Complement-dependent binding of DC was most pronounced in the case of FeO-DEX. Macrophages (CD11b^+^CD11c^−^) within the same cultures displayed similar binding patterns (Figure S8A). Albumin NC engaged DC and non-DC at the highest level when pre-incubated with h.i. serum (Figure S8B). These observations confirm that BMDC are a suitable model to study complement-dependent NC binding.

### 2.3. CR3, but Not CR4 Is Required for Binding and Uptake of Complement-Opsonized NC by DC

To elucidate the importance of CR3 on BMDC for the engagement of complement-opsonized NC further, we employed BMDC derived from CD11b^−/−^ mice, which thereby express CR4 only. Since all types of NC that were coated with carbohydrates yielded similar results, with FeO-DEX displaying the most significant differences, we used only this type of NC throughout the following experiments.

**Figure 3:**
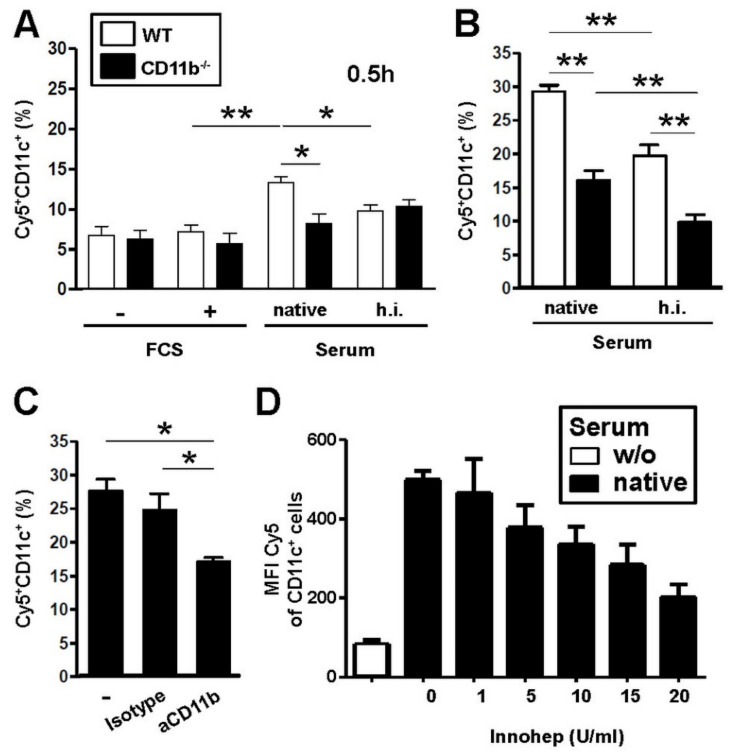
Binding of native serum-pre-incubated solid iron oxide core (FeO-DEX) to BMDC depends on complement activation and CR3 engagement. (**A**,**B**) BMDC differentiated from bone marrow progenitors derive from wild type (WT), and CD11b^−/−^ mice were incubated with differentially pretreated (left untreated (w/o), pretreated with native or heat-inactivated (h.i.) serum) FeO-DEX as indicated. (**A**) BMDC were incubated with non-pretreated FeO-DEX (BMDC kept in parallel in fetal calf serum (FCS) free (−) or standard culture medium (+) and with native and h.i. serum-pre-incubated FeO-DEX (BMDC kept in FCS-containing media) as indicated. After 0.5 h, frequencies of Cy5^+^ BMDC (CD11c^+^ cells) were assessed by FACS analysis (mean ± SEM of 3 experiments). (**B**) WT and CD11b^−/−^ BMDC were mixed and incubated for 4 h with FeO-DEX pre-incubated with native or h.i. serum. Frequencies of Cy5^+^ BMDC were assessed by FACS analysis (mean ± SEM of 3 experiments). (**C**) WT BMDC were pretreated in parallel with blocking anti-CD11b and a corresponding isotype control antibody, followed by incubation with native serum-pre-incubated FeO-DEX applied 45 min later. Frequencies of Cy5^+^ BMDC were assessed after 3 h by FACS analysis. (**D**) Native serum was pre-incubated in parallel with increasing doses of complement blocking heparin (Innohep) for 10 min. FeO-DEX were added, and samples were incubated for 1 h. Pretreated FeO-DEX were applied to WT BMDC, and after 4 h, Cy5 MFI of DC (CD11c^+^) were monitored (mean ± SEM of 3 experiments). (**A**–**C**) Statistical differences between groups are indicated (one-way ANOVA, Tukey’s test). * *p* < 0.05, ** *p* < 0.01.

Untreated FeO-DEX applied to BMDC kept in fetal calf serum (FCS) free culture medium or in normal culture medium displayed only minor cell binding (Figure 3A), which indicates that the presence of FCS in culture media has no effect on the binding of FeO-DEX in this regard. However, when FeO-DEX were pre-incubated with native mouse serum, only WT, but not CR3-deficient BMDC engaged FeO-DEX to a higher extent. As expected, pretreatment of FeO-DEX with h.i. serum did not result in significantly enhanced binding to either BMDC population. Complement-dependent binding of FeO-DEX to BMDC via CR3 was evaluated by mixing WT and CR3-deficient BMDC, followed by incubation with differentially pretreated FeO-DEX. In subsequent flow cytometric assays, BMDC were differentiated according to the dual expression of CR3 and CR4 (WT) versus CR4 only (CD11b^−/−^). Interestingly, CD11b^−/−^ DC displayed lower FeO-DEX engagement not only in the case of NC pretreatment with native but also h.i. serum, which may suggest the involvement of CR3 in binding of FeO-DEX with a complement-deficient protein corona (Figure 3B). To rule out indirect effects of CR3 on FeO-DEX binding, e.g., due to differential expression of other endocytic surface receptors by CD11b^−/−^ as compared to WT BMDC, the latter were incubated with a CD11b (and thereby CR3) blocking antibody. Blockade of CD11b, but not the application of a corresponding isotype control antibody, significantly reduced binding of FeO-DEX pretreated with a native mouse (Figure 3C). Heparin has been demonstrated to inhibit the function of various complement factors [26]. Therefore, as an alternative approach to inhibit complement activity in serum, instead of mild heat treatment, serum was pretreated with a clinically applied heparin formulation (Innohep) prior to the addition of FeO-DEX for protein corona formation. Subsequent binding assays showed decreased binding of FeO-DEX pre-incubated with Innohep-pretreated serum to BMDC in a dose-dependent manner (Figure 3D). This finding confirmed the role of carbohydrate-dependent complement activation on the surface of FeO-DEX as a prerequisite for CR3 engagement.

**Figure 4:**
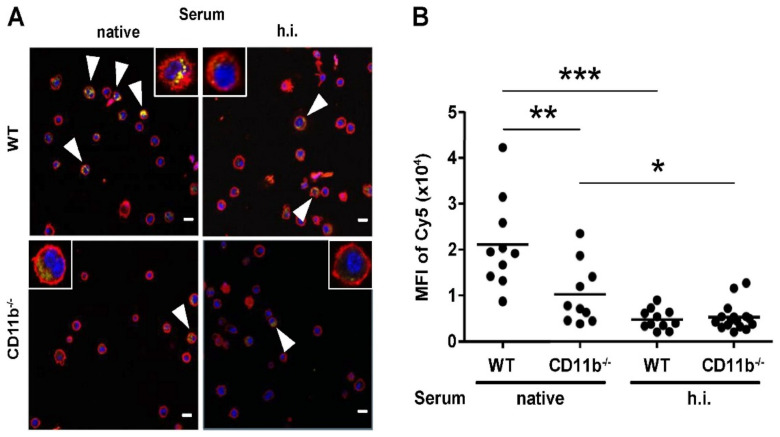
Complement activity and CR3 expression are required for uptake of FeO-DEX by BMDC. In parallel assays, WT and CD11b^−/−^ BMDC were incubated with FeO-DEX pretreated with native and h.i. serum, respectively. After 3 h, samples were cytospun, incubated with anti-CD11c antibody (red), and DAPI for nuclear staining (blue). Samples were assessed by confocal laser scanning microscopy (CLSM). Cy5 fluorescence of FeO-DEX is given in yellow. (**A**) Representative CLSM pictures. Inlets: single-cells. Arrowheads indicate Cy5-positive cells. Bar length: 10 µm. (**B**) Quantification of intracellular Cy5 intensities of Cy5^+^ BMDC (mean ± SEM of each 10 CD11c^+^ BMDC). Statistical differences between groups are indicated (one-way ANOVA, Tukey’s test). * *p* < 0.05, ** *p* < 0.01, *** *p* < 0.001.

Confocal laser scanning microscopy (CLSM) confirmed that complement-opsonized FeO-DEX were internalized to a higher extent by WT than by CD11b^−/−^ BMDC (Figure 4). When h.i. serum was used for FeO-DEX pretreatment, NC were scarcely internalized by either BMDC population. Altogether, these observations show that the enhanced binding/uptake of FeO-DEX that were pre-incubated with native mouse serum by BMDC depends on complement activation and CR3 expression.

### 2.4. Binding of Complement-Coated NC Interferes with DC Activation

**Figure 5:**
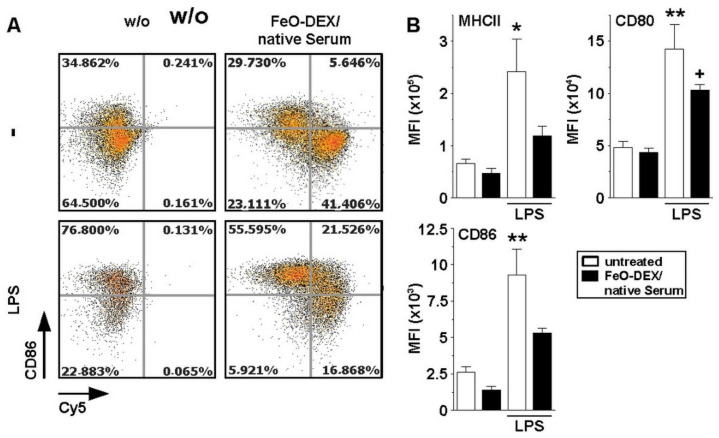
Engagement of CR3 by serum-opsonized FeO-DEX attenuates upregulation of activation markers in response to stimulation. BMDC were incubated with native serum-pre-incubated FeO-DEX. Aliquots were treated with LPS (100 ng/m) applied 1 h later. On the next day, the expression of DC activation markers (MHCII, CD80, CD86) was assessed by FACS analysis. (**A**) Representative FACS plots showing CD86 expression (gating strategy described in Figure S6). (**B**) Quantification of activation marker expression (MFI) of CD11c^+^ BMDC (mean ± SEM of 3 experiments). Statistical differences versus *untreated and ^+^FeO-DEX/native serum are indicated (one-way ANOVA, Tukey’s test). *^,+^ *p* < 0.05, ** *p* < 0.01.

Engagement of DC by immune complexes via CR3 has been shown to affect their activation state [27]. To delineate the potential effects of complement-opsonized FeO-DEX in this regard, BMDC were incubated with native serum-pretreated FeO-DEX. In parallel settings BMDC were stimulated with LPS after NC application. On the next day, the expression of activation markers was assessed. As depicted in Figure 5, FeO-DEX-positive BMDC were characterized by a somewhat lower expression of MHCII and the costimulator CD86 as compared to untreated BMDC. Further, the application of LPS as a genuine TLR4 ligand resulted in profound upregulation of either activation marker in BMDC. This effect was markedly attenuated in the case of samples incubated with complement-opsonized FeO-DEX. This result shows that engagement of CR3 on BMDC by serum-opsonized FeO-DEX diminished their responsiveness towards stimulation.

### 2.5. Among B Cells, FeO-DEX Are Highly Engaged by B-1 Cells

**Figure 6:**
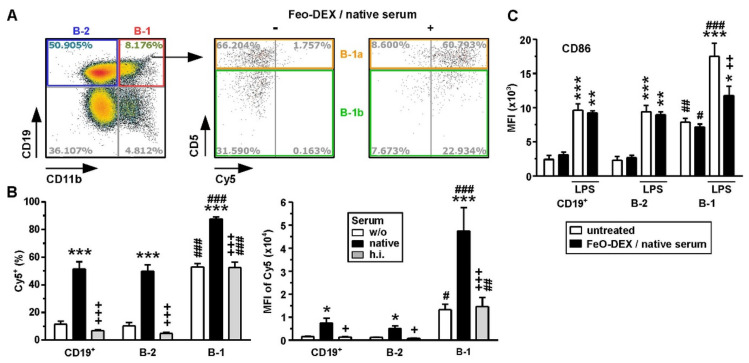
B-1 cells engage FeO-DEX to a higher extent than B-2 cells but are affected in their activation state. Spleen cells were incubated over-night with differentially pretreated (left untreated (w/o), pretreated with native or heat-inactivated (h.i.) serum) FeO-DEX. (**A**) FACS plot identifying splenic B-1 cells that co-express CD19 and CD11b (left panel) and delineating CD5^+^ B-1-a and CD5- B-1b cells, which engage native serum-pre-incubated FeO-DEX (right panel). Plots are representative of 4 experiments. (**B**) Frequencies of Cy5^+^ (left panel) and MFI of Cy5 (right panel) of B cell populations (total B cells (CD19^+^), B-2 cells (CD11b-CD19^+^) and B-1 cells (CD11b^+^CD19^+^)). Data denote the mean ± SEM of 3 experiments. Statistical differences versus *w/o, +native serum, #B-1 versus B-2 (corresponding group) are indicated (one-way ANOVA, Tukey’s test). (**C**) Quantification of CD86 activation marker expression (MFI) of B cell populations (see above) after over-night incubation with native serum-pretreated FeO-DEX (mean ± SEM of 3 experiments). Statistical differences versus *according to the group at unstimulated state, ^#^B-1 versus B-2 (corresponding group) and ^+^LPS-stimulated are indicated (one-way ANOVA, Tukey’s test). (**B**,**C**) *^,+,#^ *p* < 0.05, **^,++,##^ *p* < 0.01, ***^,+++,###^ *p* < 0.001.

We have previously demonstrated that complement-opsonized FeO-DEX bound splenic B cells via CR1/2 [7]. B-1 cells comprise a minor B cell population, which, besides CR1/2, also expresses CR3 [28]. Therefore, we asked whether B-1 cells, due to co-expression of both complement receptors, would bind FeO-DEX to a higher extent as compared to conventional B-2 cells. To this end, murine spleen cells were incubated with differentially pretreated FeO-DEX, and their binding to B cell subpopulations was assessed by flow cytometry. In our experiment, up to 8% of splenic CD19^+^ B cells constituted B-1 as deduced from co-expression of CR3 (Figure 6A, left panel). B-1 cells are phenotypically subdivided into CD5^+^ B-1a and CD5^−^ B-1b cells. Both B-1 subpopulations readily engaged FeO-DEX as assessed after over-night incubation (right panel). B-1 cells bound directly applied FeO-DEX at a much higher extent than B-2 cells, both in terms of frequency (Figure 6B, left panel) and overall MFI (Figure 6B, right panel). Pretreatment of FeO-DEX with native serum enhanced their binding to either B cell population. This effect was abolished when FeO-DEX were pretreated with h.i. serum. Similar results were also obtained after 3 h incubations (Figure S9).

Due to our findings of an inhibitory effect of FeO-DEX engagement of DC with regard to their response towards subsequent stimulation, we asked for corresponding effects in the case of B cells. After incubation with native-serum-pretreated FeO-DEX, FeO-DEX-binding B-1 and B-2 cells displayed no alterations in the case of costimulator CD86 (Figure 6C) expression. Both B cell subpopulations were characterized by elevated CD86 expression after LPS stimulation. B-2 cells stimulated with LPS after the onset of incubation with serum-pretreated FeO-DEX displayed comparable CD86 expression levels as non-incubated samples. In contrast, FeO-DEX-positive B-1 cells were characterized by diminished upregulation of CD86 in response to subsequent stimulation with LPS. Therefore, binding of serum-pretreated FeO-DEX may exert inhibitory effects on activation marker expression by B-1 cells. Taken together, these findings show that B-1 cells, in general, engage FeO-DEX to a higher extent than B-2 cells and that binding of complement-opsonized FeO-DEX affected their activation state, which was not observed for B-2 cells.

**Figure 7:**
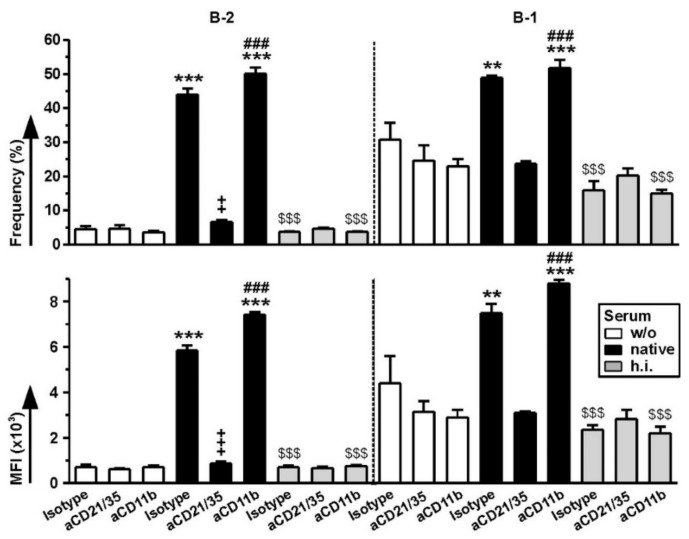
B-1 cells engage complement-opsonized FeO-DEX via CR1/2, but not CR3. Spleen cells were pretreated in parallel with antagonistic anti-CD21/CD35 (CR1/2), anti-CD11b (CR3) and an according isotype control antibody (each 5 µg/mL), followed by incubation with differentially pretreated FeO-DEX (left untreated (w/o), pretreated with native or heat-inactivated (h.i.) serum) applied 45 min later. Frequencies of Cy5^+^ cells (upper panel) and MFI of Cy5 (lower panel) of B-2 (left panel) and B-1 (right panel) cells, identified as CD11b^−^CD19^+^ (B-2 cells) and CD11b^+^CD19^+^ (B-1 cells) were assessed 3 h after the onset of incubation with FeO-DEX (mean ± SEM of 3 experiments). Statistical differences * versus w/o and ^$^ versus native serum group (treated with the same antibody), ^+^ versus isotype and ^#^versus aCD21/35 (within same group) are indicated (one-way ANOVA, Tukey’s test). *^,+,#^ *p* < 0.05, **^,++,##^ *p* < 0.01, ***^,+++,###,$$$^ *p* < 0.001.

Finally, we asked for the relative importance of CR1/2 and CR3 as expressed by B-1 cells for the binding of differentially pretreated FeO-DEX. To this end, spleen cells were pretreated with antagonist antibodies for either CR, followed by incubation with differentially pretreated FeO-DEX for 3 h. In contrast, binding of FeO-DEX left untreated or pretreated with h.i. serum to B-2 and B-1 cells was not affected by either antagonistic antibody, binding of serum-pretreated FeO-DEX to either B cell subpopulation in the case of CR1/2 blockade (Figure 7). Similar observations were made after over-night incubation (Figure S10A). Interestingly, blockade of CR3 yielded no such effect on B-1 and B-2 cells but strongly reduced Cy5 intensities of CD11b-expressing non-B cells (Figure S10B). These observations suggest that the elevated capacity of B-1 cells to bind (complement-opsonized) FeO-DEX does not depend on CR3 but CR1/2 only.

## 3. Discussion

It is well established by now that in vivo, a protein corona may be formed around NC, which may significantly affect cellular interaction, and thereby its targeting and drug delivery properties. Aside from “passive” protein adsorption onto the NC surface [2], surface structures of the NC may also be recognized by the innate immune system, as shown by us and others for carbohydrate-coated NC, which triggered the lectin-dependent complement pathway [7].

Here we show that CR3-expressing cell types within the spleen, including myeloid cell types [9], engaged complement-opsonized NC to a higher extent as compared to NC applied directly or after pretreatment with h.i. serum lacking complement activity. We further showed that CR3 is necessary for binding and uptake of complement-opsonized NC by BMDC, whereas CR4, which in mouse constitutes a pan-DC lineage marker [29], is dispensable. It is conceivable that in the case of cDC2, CR3 may largely contribute to the binding of (complement-opsonized) NC. However, in the case of cDC1 and pDC, which lack CR3, other receptors may play a role in NC binding. Additional studies are necessary to identify contributing receptors.

Besides DEX and starch also other carbohydrates like chitosan [30] were reported to trigger complement activation, which is of considerable importance given the ongoing interest in chitosan-based and -coated NC for biomedical applications [31]. Aside from carbohydrates also other surface properties may trigger complement activation as reported for positively charged poly(lactic-co-glycolic) acid (PLGA) based NC [32], another type of NC frequently used in preclinical [33] and clinical [34] studies. Therefore, in vivo complement opsonization of NC may contribute to their biodistribution in numerous cases.

CR3 may play an important role in the binding and uptake of complement-opsonized NC in the case of myeloid cells and NK cells. CR3 may also facilitate the binding of NC via other constituents of their protein corona due to its rather broad substrate affinities, including numerous serum factors [9]. In agreement, polystyrene microparticles coated with bovine serum albumin, fibronectin or fibrinogen showed stronger uptake by WT as compared to CD11b^−/−^ MAC [35]. Above, adsorption of fibrinogen onto negatively charged poly(acrylic acid)-conjugated gold NC resulted in its unfolding, which in turn enhanced its binding to MAC via CR3 [36]. Superparamagnetic iron oxide nanoparticles, which are clinically employed for resonance imaging of atherosclerotic plaques [37], when tested in vitro, engaged CR3 as delineated using CD11b-blocking antibodies [38]. However, it remains unclear whether, under these circumstances, constituents of the protein corona, which is inevitably formed by contact with FCS in cell culture media [39], may have served as CR3 ligand(s).

Anyway, that study also highlighted that the activation state of CR3 might contribute to the extent of NC engagement. In this regard, it is noteworthy that CR3 can exhibit different conformation states, which in turn may affect its binding affinity for substrates [20]. The conformation state of CR3 is dynamically regulated by distinct mediators and signaling pathways as well as ligands [40,41] in a cell type-specific manner [42]. Insofar, both the cellular microenvironment and the character of the CR3 engaging ligand may contribute to the extent of its binding to CR3-expressing cells.

In general, CR3 may also serve as an inhibitor of DC activation since DC deficient for CD11b [43] and CD18 [19] displayed hyper-activation in response to stimulation with TLR ligands. Similar observations were made for MAC [19,44]. We observed that BMDC, which bound complement-opsonized FeO-DEX were characterized by diminished upregulation of activation markers in response to subsequently applied LPS. This result corroborates with previous studies that reported inhibitory effects of CR3 triggering on DC, e.g., by complement-opsonized apoptotic cells, shown to promote self-tolerance [45,46]. In the case of infection, however, opsonized pathogens, which are frequently recognized and internalized by CR3, yield activation of DC (and other types of APC) due to co-delivered pathogen-derived molecular patterns that act as danger signals [47] and thereby override the inherent inhibitory effect of CR3 triggering.

Danger signals, as exemplified, e.g., for LPS, were reported to increase CR3 surface expression [48,49]. Therefore, it is conceivable that NC intrinsically exerts adjuvant activity, as, for example, poly (lactic-co-glycolic acid) (PLGA) [50], and/or deliver a danger signal intended to serve as an adjuvant (e.g., immunostimulatory TLR ligands like unmethylated CpG motifs [51]) into a myeloid cell may in the course of cell activation also mediate the acquisition of a high-affinity state of CR3 and overall elevated CR3 expression. By these mechanisms, CR3-dependent binding and uptake of applied NC may be increased. However, at later time points after the onset of activation, in the case of professional APC like cDC, internalization of the material may cease as a consequence of the functional switch of the activated APC to emigrate towards secondary lymphoid organs to present (pathogen-) derived antigen to T cells [52].

Our studies also showed that within the splenic B cell population, especially B-1 cells, characterized by CR3 expression, engaged FeO-DEX at a stronger extent than “conventional” B-2 cells. This difference was apparent both in the case of non-pretreated FeO-DEX as well as NC pretreated with native or h.i. serum. Somewhat surprisingly, our blocking studies, on one hand, suggest that CR3 plays no role in the engagement of either type of differentially pretreated FeO-DEX, which could be explained by a low-affinity conformation state of CR3 [40,41]. On the other hand, CR1/2 was essential for binding of serum-pretreated FeO-DEX, both in the case of B-1 as well as B-2 cells, which corroborates our previous findings [7]. Similar to BMDC, engagement of complement-opsonized FeO-DEX affected surface expression of CD86 of B-1 cells. Although CR3 played no role in the binding of FeO-DEX, we cannot rule out a contribution of CR3 as a signaling adaptor to inhibit the state of activation of B-1 cells after contact with FeO-DEX. Further studies are required to delineate which signaling adaptors are important in this regard.

B-1 cells reside predominantly in the peritoneum and pleural cavities [53]. As an early response to infection, B-1 cells via interferon I-induced activation of CR3 migrate towards lymphatic organs [54] and are activated further by pathogen-derived stimuli [17]. Deficiency of CD11b (and thereby CR3) was reported to result in hyper-activation and prolonged survival of B-1 cells [55], as also has been observed for myeloid cell types [43,44]. B-1 cells comprise two subpopulations, which are phenotypically differentiated according to co-expression of CD5 (CD5^+^: B-1a, CD5^−^: B-1-b) and display a different ontogeny [16]. In our studies, B-1a and B-1b engaged FeO-DEX at comparable levels. Both B-1 subpopulations exert primarily innate immune functions to eradicate pathogens by producing (natural) IgM antibodies [56] and exert phagocytosis [17] but also serve as APC that induces (primary) T cell responses [28,57]. Altogether, B-1 cells, due to their innate-like functional properties, significantly contribute to early immune responses in viral [58] and parasite [17] infections. Consequently, B-1 cells have been ascribed strong therapeutic potential, as confirmed in a number of adoptive transfer studies [59].

Conventional antigen-specific B-2 cells play an important role in the later course of infection by producing antibodies that opsonize pathogens [60] and bind infected cells, which de novo express pathogen-specific proteins on their surface, resulting in antibody-dependent cellular cytotoxicity, cell phagocytosis and complement-dependent cytotoxicity [61]. The same mechanisms are exploited by numerous clinically applied monoclonal antibodies designed to label malignant cells [62]. We exploited the intrinsic, complement-mediated binding of FeO-DEX to splenic B cells to shift T helper cell type 2 (Th2)-biased humoral responses towards Th1 [7] using CpG ODN as an adjuvant [63]. In therapeutic settings, vaccination of mice with FeO-DEX conjugated with ovalbumin (OVA) as a model antigen and CpG ODN prevented allergic reactions by inhibiting IgE production [7]. It is conceivable that by this approach, besides B-2 cells, also B-1 cells, as well as myeloid cells, may have been addressed.

To date, most NC-focused immunotherapeutic strategies aimed to induce adaptive immune responses to target DC [64], which at an activated state constitute the most potent APC population, able to induce primary T cell responses [22]. In contrast, only a few studies have issued active B cell targeting [65]. To the best of our knowledge, the engagement of B-1 cells by NC has not been described before. In light of the important role of B-1 cells for innate as well as adaptive immunity, further studies should also take into account the potential interaction of this cell type with NC and the consequences concerning the state of activation of NC-binding B-1 cells.

Altogether, our results confirm that the design of NC for biomedical purposes needs to take into account the possibility of recognition of surface structures like carbohydrates by the complement system, resulting in complement opsonization and predominant interaction with CR-expressing leukocytes. Above, in some cases, an NC may trigger the alternative complement pathway due to its “foreign” surface as recently demonstrated for silica particles [66], and the classical complement pathway in the case (natural) antibodies that bind NC surface structures, resulting in recognizable exposed Fc ends [67]. Of note, activation of the classical complement pathway also has been demonstrated for NC coated with poly(2-methyl-2-oxazoline) (PMOXA), actually intended to prevent serum protein adsorption [68].

Aside from complement activation also other constituents of the protein corona may yield targeting, e.g., of CR3, like denatured fibrinogen [36], and of SR-A [69]. With regard to the latter, we observed no contribution of SR-A to cellular binding of differentially pretreated carbohydrate-coated types of NC as demonstrated for FeO-DEX. It remains a pressing issue, which factors governed binding, especially of (h.i.) serum-pretreated carbohydrate-coated NC to liver NPC. It is tempting to speculate that besides the involvement of CR3 and CR4, respectively, other receptors may contribute to NC engagement. The latter holds true also for LSEC since this cell type is not known to express any CR [24] but showed elevated binding of carbohydrate-coated NC pretreated with native/h.i. serum. Since we observed no involvement of SR-A in this regard, and due to the fact that most types of NC after systemic application strongly accumulate in the liver, further studies are paramount to identify the contributing receptors. By now, a number of strategies have been developed to minimize unwanted cellular interaction of NC, including the use of PEG [70] and dysopsonic proteins [4] to minimize protein adsorption [4,70], and conjugation with CD47 serving as a “do not eat me” signal to reduce uptake by myeloid cell types [71].

In general, our study confirms the necessity to include serum in in vitro studies on cellular interaction of NC to mimic somewhat more closely the behavior of NC in vivo on a cellular level, as shown by us [7] and others [72], and to use primary (immune) cells for testing. More specifically, most in vitro studies on NC binding properties, which are performed as a prerequisite for subsequent in vivo testing, are performed using cell lines or primary immune cells that are rather easily accessible, like peripheral blood mononuclear cells (human) or splenic immune cells (mouse). However, in light of the frequent finding of strong NC accumulation in the liver in vivo, it may be of advantage to include liver NPC as well [24].

**Figure 8:**
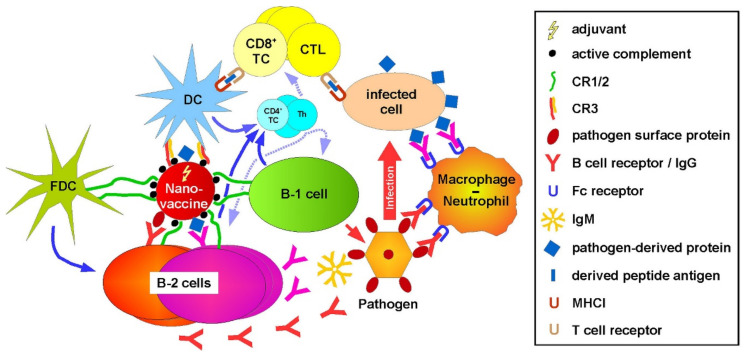
Nano-vaccines coated with active complement may concomitantly induce cellular and humoral immune responses. A nano-vaccine decorated with active complement may address B cells (B-1, B-2) and follicular dendritic cells (FDC) via CR1/2 and (conventional) DC via CR3. In the case of B-2 cells, antigen-specific internalization may be achieved by decoration with a pathogen-specific protein binding the B cell receptor. Codelivery of an adjuvant will result in the production of antigen-specific antibodies (B-2), which opsonize either the pathogen or the infected cells. Exposed Fc parts can trigger the classical complement pathway and directly bind Fc receptors. Antibody and/or complement-opsonized pathogens and infected cells are phagocytosed by myeloid cell types. Uptake of the nano-vaccine by B-1 cells may result in enhanced production of IgM and phagocytosis of pathogens via yet unknown receptors. Stimulated DC activates antigen-specific CD8^+^ T cells, yielding cytotoxic T lymphocytes (CTL). CTL, in turn, kills infected cells that present the corresponding antigen. Full activation of CD8^+^ T cells and B-2 cells requires help by activated CD4^+^ T cells, called T helper cells (Th). These are stimulated by activated antigen-presenting cells, namely DC and B cells (B-1, B-2). FDC facilitates sustained B cell responses.

With regard to a translational perspective, our results suggest that complement-opsonized NC could be a suitable vaccine platform to induce a broad cellular and humoral immune response, similar to pathogens that are recognized by the complement system [73]. As depicted in Figure 8, such nano-vaccines, co-delivering pathogen- or tumor-specific antigen and adjuvant, may address on one hand especially cDC2 via CR3, which at activated state stimulate antigen-specific CD4^+^ Th [74] and CD8^+^ T cells [75]. The latter yield cytotoxic T lymphocytes that recognize and kill infected or malignant somatic cells [76]. On the other hand, corresponding nano-vaccines may target as well B cells via CR1/2, as shown previously by us [7]. In the case of B-2 cells, antigen specificity may be achieved in the case the nano-vaccine is decorated with a native protein antigen, which allows for concomitant binding of the B cell receptor (BCR). In fact, it has been demonstrated that the presence [77] and triggering [78] of CR1/2 on B cells is required to mount sustained antigen-specific antibody production. In the case of a pathogen-specific nano-vaccine, the candidate antigen would be a pathogen-specific surface protein in order to induce neutralizing antibodies [79]. Alternatively (or in addition), a pathogen-derived protein, which would be apparent on the surface of infected cells [80], would be a suitable antigen, inducing the production of antibodies that recognize infected cells, and trigger classical complement activation [81] or antibody-dependent cytotoxicity [82]. A nano-vaccine applied for tumor therapy should be decorated with a tumor-associated/specific surface protein in order to obtain antibodies that recognize malignant cells and confer antibody-mediated cytotoxic effects [83,84]. Targeting of B-1 cells by a nano-vaccine via CR1/2 may result in a profound generation of IgM antibodies [56]. In addition, B-1 cells [17,28] and myeloid cells [85], which are targeted via CR-3, may exert on one hand elevated innate responses, like phagocytosis, and on the other hand, acquire APC activity, and thereby contribute to the overall induction of cellular immune responses. DC [86] and B cells [87] that engage the nano-vaccine will also induce Th cells, which in turn activate CD8^+^ T cells [88] and B cells [89].

Follicular dendritic cells (FDC) contribute to affinity maturation and memory B cell induction and survival [90] by long-term storage of complement-opsonized immune complexes [90] that are internalized via CR1/2 [91]. We previously observed binding and uptake of FeO-DEX by FDC in vivo [7], suggesting the involvement of CR1/2. It remains to be shown whether direct addressing of FDC by a complement-opsonized nano-vaccine may yield beneficial effects concerning B cell affinity maturation and B cell memory.

## 4. Materials and Methods

### 4.1. Materials

Fluorescent-labeled types of NP were obtained from micromod (Rostock, Germany) or from Miltenyi Biotech (Bergisch Gladbach, Germany). The characteristics of the various types of NP are listed in Table S1. Mouse serum was purified from whole blood (C57BL/6) by centrifugation using Z Serum Sep columns (Greiner Bio-One, Frickenhausen, Germany) and was quantified in a NanoDrop 2000 (Thermo Fisher, Waltham, MA, USA). Protein contents were about 30 mg/mL (Nanodrop 2000; Thermo Fisher). Aliquots of native mouse serum were heat-inactivated (56 °C, 30 min). Fucoidan, dextran sulfate, chondroitin sulfate, polyinosinic acid and polycytidylic acid (all from Sigma-Aldrich, Deisenhofen, Germany) were used to assess the contribution of SR-A for FeO-DEX binding. The heparin formulation Innohep (Leo Pharma, Neu-Isenburg, Germany) was used to inhibit the complement activity of mouse serum. Antibodies used for FACS analysis are listed in Table S2. PE/Cy7-labeled anti-CD11c antibody (clone N418) and DAPI (nuclear staining) used for CLSM analysis were purchased from Thermo Fisher. To assess the role of distinct CR for NC binding, samples were incubated with antibodies that block CR1/2 (anti-CD21/CD35; clone 7G6; BD Biosciences, Franklin Lakes, NJ, USA) and CR3 (anti-CD11b; clone M1/70; BioLegend, San Diego, CA, USA), respectively. In parallel, a corresponding isotype control antibody (Thermo Fisher) was employed.

### 4.2. Mice

C57BL/6 mice and CD11b^−/−^ mice on a C57BL/6 background (B6.129S4-Itgam^tm1Myd^/J) were bred and maintained in the Central Animal Facility of the Johannes Gutenberg-University Mainz under specific pathogen-free conditions on a standard diet according to the guidelines of the regional animal care committee. Female mice (6–12 weeks) were used throughout all experiments. The “Principles of Laboratory Animal Care” (NIH publication no. 85-23, revised 1985) were followed. Mice were sacrificed for organ retrieval according to § 4(3) TierSchG.

### 4.3. Immune Cells

Liver nonparenchymal cells (NPC) were isolated using a liver dissociation kit and the gentle MACS dissociator (both Miltenyi Biotec) as recommended by the manufacturer with some modifications. In brief, after liver dissociation, cells were resuspended in 1 mL cold HBSS buffer (w/o Ca^2+^ and Mg^2+^). The cell suspension was mixed with a double volume of freshly prepared 30% HistoDenZ (Sigma-Aldrich) and overlaid with 1 mL of cold HBSS buffer. After centrifugation (1500× *g*, 4 °C, w/o break), the cell containing interphase was retrieved and washed. Erythrocytes were lysed using a hypotonic buffer.

Spleen cells were isolated using a 40 µM cell strainer (Greiner Bio-One) to obtain a single-cell suspension. In parallel settings, spleen cells were resuspended in medium (IMDM, 2 mM L-glutamine, 100 U/mL penicillin, 100 µg/mL streptomycin (all from Sigma-Aldrich, Deisenhofen, Germany), and 50 µM ß-mercaptoethanol (Roth, Karlsruhe, Germany)) containing 5% FCS (PAN).

Bone marrow cells (2 × 10^5^/mL) were seeded in 12-well cell cluster plates (Greiner Bio-One) in an IMDM-based culture medium (see above) supplemented with 10 ng/mL recombinant murine GM-CSF (R&D Systems, Wiesbaden, Germany). Culture media was replenished on days 3 and 6 of culture.

### 4.4. NP Binding Studies

Aliquots of the different NP were pre-incubated in parallel with equal volumes of native or heat-inactivated mouse serum for 1 h at 37 °C in low protein binding 0.5 mL reaction tubes (Greiner Bio-One). Afterward, untreated and differentially pretreated NP were applied at concentrations evaluated in corresponding pre-experiments (see Table S1) to BMDC differentiated in 12-well cell cluster plates and to the spleen cells and liver NPC kept in sterile FACS tubes (each 2 × 10^6^/500 µL). Three hours after the onset of incubation, samples were harvested and subjected to flow cytometric analysis. In some cases, cells were harvested after over-night incubation. In some cases, samples were stimulated overnight with LPS (100 ng/mL; Sigma-Aldrich). The relevance of distinct CR for NC binding was assessed using CR-blocking antibodies and a corresponding isotype control antibody (each 5 µg/mL; see 4.1. Materials) applied 45 min prior to administration of NC.

The contribution of class A scavenger receptors for binding of FeO-DEX was tested using various pharmacological inhibitors. To this end, cells were pre-incubated with fucoidan (25 µg/mL), dextran sulfate and chondroitin sulfate (each 25 µg/mL), and polyinosinic acid and polycytidylic acid (each 12.5 µg/mL) for 1 h prior to application of differentially pretreated FeO-DEX.

### 4.5. Flow Cytometry

After incubation with NP, cells were washed in staining buffer (PBS/2% FCS), and Fcγ receptors were blocked by applying rat anti-mouse CD16/CD32 antibody (clone 2.4G2; 15 min, room temperature). Afterward, samples were incubated (20 min, 4 °C) with distinct sets of fluorescence-labeled antibodies as indicated in Table S2. Subsequently, samples were incubated with FVD (eFl450 or eFl780) to delineate dead cells. Antibodies were purchased from BD Biosciences, Thermo Fisher or BioLegend. FVD was obtained from Thermo Fisher. Samples were analyzed on an Attune NxT flow cytometer (Thermo Fisher). Data were analyzed using FlowJo software (FLOWJO, Ashland, OR, USA).

### 4.6. CLSM

BMDC (d8 of culture) were incubated with FeO-DEX as indicated, washed with FACS buffer (PBS, 1% FCS, 0.5 mM EDTA), and transferred onto chamber slides (IBIDI, Martinsried, Germany). Cells were incubated with anti-CD11c-PE/Cy7 antibody and DAPI for nuclear staining. Confocal microscope: LSM510-UV (Zeiss, Jena, Germany).

### 4.7. Statistical Analysis

Statistical analysis was performed using GraphPad Prism Software v5.0 (GraphPad Software Inc., San Diego, CA, USA). Results were expressed as mean ± standard error of the mean (SEM). Differences among groups were tested by one-way ANOVA, followed by post hoc Tukey’s test, assuming significance at *p* < 0.05.

## Data Availability

All relevant data are included within the manuscript.

## Supplementary Materials

Supplementary Materials can be found at https://www.mdpi.com/1422-0067/22/6/2869/s1.

## Abbreviations

APC	Antigen-presenting cell
BM	Bone marrow
CR	Complement receptor
cDC	Conventional dendritic cell
CLSM	Confocal laser scanning microscopy
DC	Dendritic cell
FCS	Fetal calf serum
FDC	Follicular dendritic cells
LSEC	Liver sinusoidal endothelial cell
MAC	Macrophage
NC	Nanocarrier
NPC	Nonparenchymal cell
NK	Natural killer cell
ODN	Oligodeoxynucleotides
OVA	Ovalbumin
pDC	Plasmacytoid DC
PMN	Polymorphonuclear cell
SPION	Superparamagnetic iron oxide nanoparticles
SR-A	Scavenger receptor class A
Th	T helper cell
TLR	Toll-like receptor
WT	Wild type
