# Supplementary material for: Complement-Opsonized Nano-Carriers Are Bound by Dendritic Cells (DC) via Complement Receptor (CR)3, and by B Cell Subpopulations via CR-1/2, and Affect the Activation of DC and B-1 Cells
Source: Int J Mol Sci. 2021 Mar 11;22(6):2869. doi: 10.3390/ijms22062869 (PMC8001596; doi:10.3390/ijms22062869)
Supplement: Supplementary file 1 [file ijms-22-02869-s001.pdf]

International Journal of
Molecular Sciences

MDPI

Supplemental Materials

Complement-Opsonized Nano-Carriers are Bound by Dendritic Cells (DC) via Complement Receptor (CR)3, and by B Cell Subpopulations via CR-1/2, and Affect the Activation of DC and B-1 Cells

Monika Bednarczyk[1], Carolina Medina-Montano[1], Frederic Julien Fittler[1,+], Henner Stege[1,+], Meike Roskamp[2], Michael Kuske[1], Christian Langer[2], Marco Vahldieck[2], Evelyn Montermann[1], Ingrid Tubbe[1], Nadine Röhrig[1], Andrzej Dzionek[2], Stephan Grabbe[1] and Matthias Bros[1,*]

[1] Department of Dermatology, University Medical Center Mainz, Langenbeckstraße 1, 55131 Mainz, Germany; m.bednarczyk.09@aberdeen.ac.uk (M.B.); gmedinam@students.uni-mainz.de (C.M.-M.); ffittler@students.uni-mainz.de (F.J.F.); Henner.Stege@unimedizin-mainz.de (H.S.); mikuske@uni-mainz.de (M.K.); monterma@uni-mainz.de (E.M.); tubbe@uni-mainz.de (I.T.); n.roehrig@uni-mainz.de (N.R.); stephan.grabbe@unimedizin-mainz.de (S.G.); mbros@uni-mainz.de (M.B.)

[2] Miltenyi Biotec GmbH, Friedrich-Ebert-Strasse 68, 51429 Bergisch Gladbach, Germany; meiker@miltenyi.com (M.R.); christianlan@miltenyi.com (C.L.); MarcoV@miltenyi.com (M.V.); Andreasd@miltenyibiotec.de (A.D.)

* Correspondence: mbros@uni-mainz.de; Tel.: +49-6131-17-9846

† These authors contributed equally to this work.

Supplemental tables

Table S1. NC properties

	NC			
Properties	BNF-STA	BNF-DEX	FeO-DEX	Albumin
Composition	Cross-linked iron oxide-starch composite	Cross-linked iron oxide-dextran composite	Iron-dextran composite	cross-linked bovine serum albumin
Diameter (nm)	100	100	63	250
Fluorescent dye[1]	RedF	RedF	Cy5	GreenF
Final concentration (µg/ml)[3]				
Liver NPC	5	50	1.25	2.5
Spleen cells	5	50	1.25	2.5
BMDC	25	10	1.25	1

[1]RedF: Excitation: 552 nm, Emission 580 nm; GreenF: 485 nm, 510 nm; Cy5: 649 nm, 666 nm; [2]assessed in pre-experiments using BMDC; [3]determined based on dose kinetic experiments using spleen cells, liver NPC and BMDC.

Table S2. Antibodies

Antibody	Fluorochrome	Clone	Marker
**Spleen cells**			
CD3	V500	500A2	T cell
CD5	PerCP	03.07.53	B-1a
CD11b	SB600	M1/70	Myeloid cell
CD11c	BV421	N418	DC
CD19	SB702	1D3	B cell
Ly6G	eFl610	1AB	PMN
NK1.1	PE-Cy7	PK136	NK cell
**Liver NPC**			
CD11c	BV421, PE-Cy7	N418	DC
CD32b	APC, PE	AT130-2	LSEC
CD45	BV711	30-F11	Leukocyte
F4/80	eFl506	BM8	Macrophage
**BMDC**			
CD11b	SB600	M1/70	Myeloid cells
CD11c	PE-Cy7 / BV421	N418	DC
CD80	PE	16-10A1	Activation

CD86	FITC	GL1	Activation
MHCII	PE-eFl610	M5/114-15.2	Activation

Supplemental figures

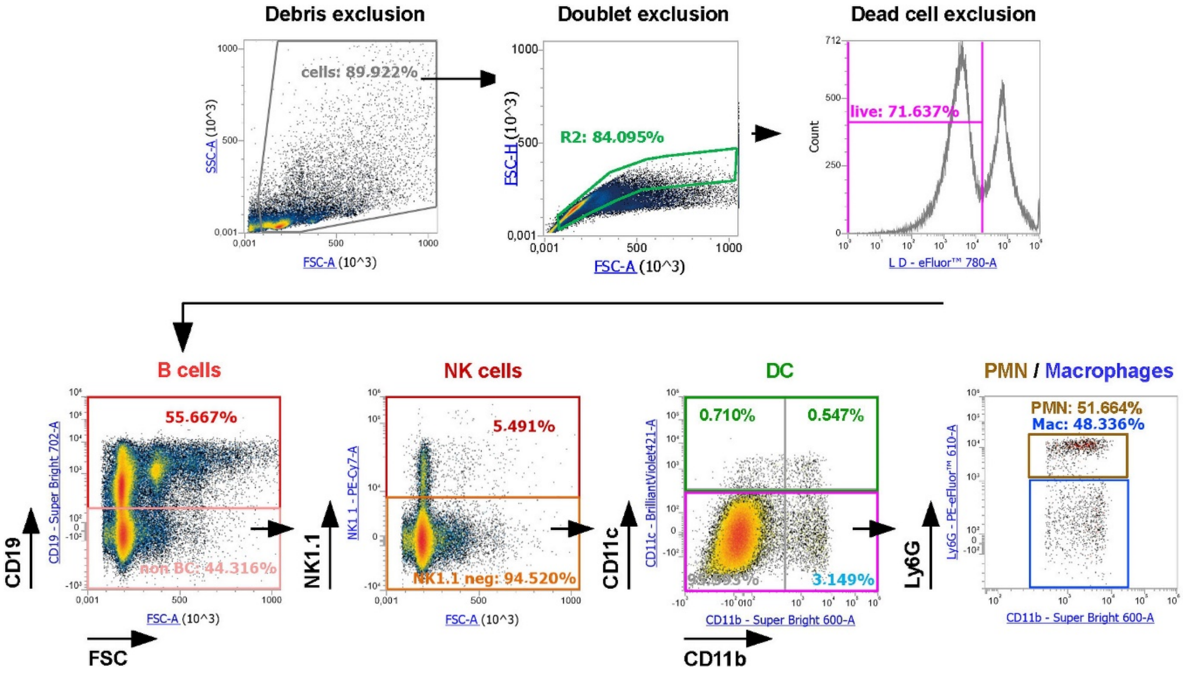

Figure S1. Gating strategy of spleen cells. After exclusion of debris, doublets and dead cells, spleen cell populations were identified sequentially according to lineage marker expression, B cells ($CD19^{+}$), NK cells ($CD19^{-}NK1.1^{+}$), DC ($CD19^{-}NK1.1^{-}CD11c^{+}$), PMN ($CD19^{-}NK1.1^{-}CD11c^{-}CD11b^{+}Ly6G^{+}$) and MAC ($CD19^{-}NK1.1^{-}CD11c^{-}CD11b^{+}Ly6G^{-}$).

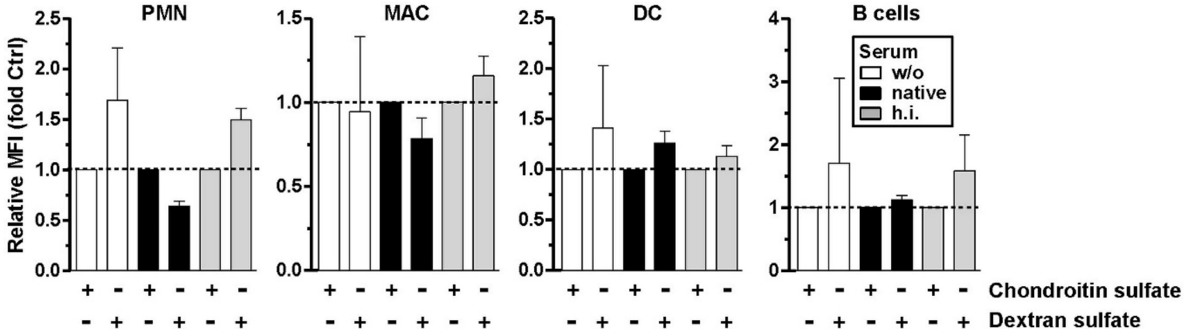

Figure S2. Scavenger receptors type A (SR-A) are not involved in binding of FeO-DEX. Spleen cells were pre-incubated in parallel with a SR-A inhibitor (dextran sulfate) and the according control agent (chondroitin sulfate, Ctrl) for 45 min. Then, samples were incubated for 3h with differentially pretreated (left untreated [w/o], pretreated with native or heat-inactivated [h.i.] serum) Cy5-labeled FeO-DEX and assayed by flow cytometry (see figure S1). Graphs denote the relative MFI of Cy5 in various splenic cell populations (mean±SD of 2 experiments).

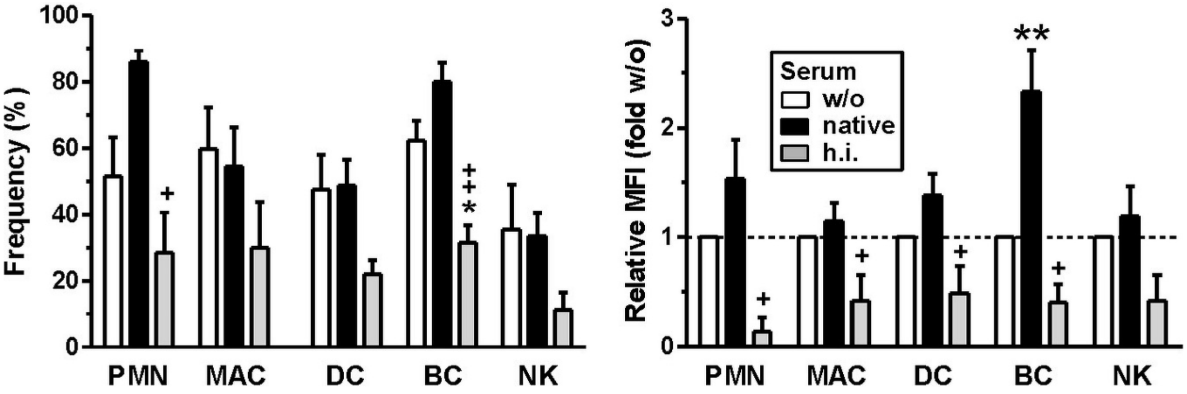

Figure S3. Albumin NC pre-incubated with h.i. serum bind most spleen cell populations at reduced extent. Differentially pretreated (left untreated [w/o], pretreated with native or heat-inactivated [h.i.] serum) GreenF-labeled albumin NC were incubated for 3h with spleen cells. The extent of NC binding to the various spleen cell populations was assessed by flow cytometry (see figures 1, S1). Graphs denote the frequencies of GreenF-positive cells (left panel) and the relative MFI of either spleen cell type (right panel) (mean±SEM of 3-4 experiments). PMN: neutrophils, MAC: macrophages, DC: dendritic cells, BC: B cells, NK: natural killer cells. Statistical differences: versus *w/o serum and +native serum (one way ANOVA, Tukey test). *,+p<0.05, **,++p<0.01.

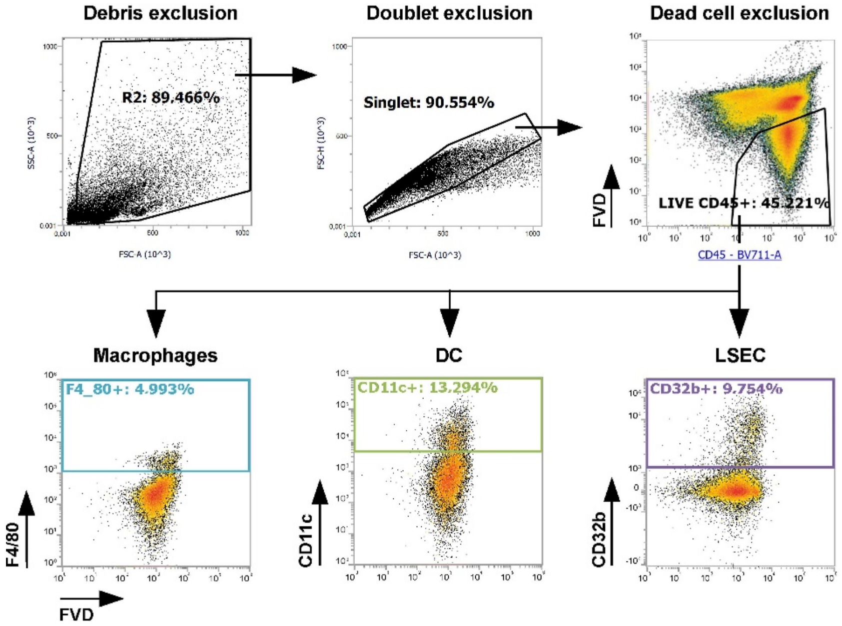

Figure S4. Gating strategy of liver NPC. After exclusion of debris, doublets and dead cells (fixable viability dye [FVD]-), CD45+ cells were differentiated according to expression of F4/80 (macrophages), CD11c (DC), and CD32b (LSEC; liver sinusoidal endothelial cells).

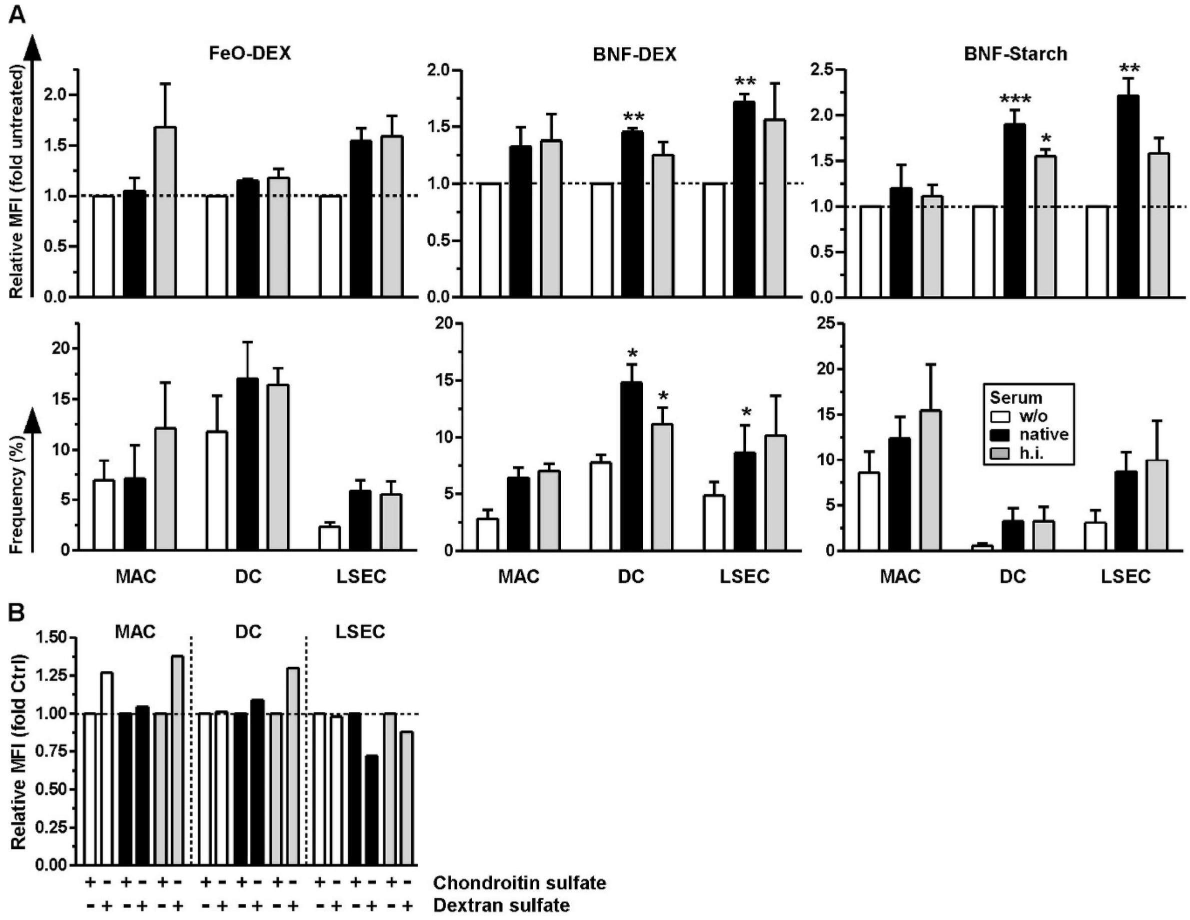

Figure S5. Binding of lectin-coated types of NC to liver non-parenchymal cells (NPC) is influenced by their protein corona. (**A**) Liver NPC were incubated with differentially pretreated (left untreated [w/o], pretreated with native or heat-inactivated [h.i.] serum) fluorescence-labeled types of NC for 3h. The extent of NC binding to macrophages (MAC), dendritic cells (DC) and liver sinusoidal endothelial cells (LSEC) was assessed by flow cytometry (see figure S4). Data denote the frequencies of NC-positive liver NPC (upper panel) and relative MFI of NC-specific fluorescence of either liver NPC population (lower panel) (mean±SEM of 3-4 independent experiments). MAC: macrophages. Statistical differences: versus *w/o serum and +native serum (one way ANOVA, Tukey test). *p<0.05, **p<0.01, ***p<0.001. (**B**) Liver NPC were pre-incubated in parallel with a SR-A inhibitor (dextran sulfate) and the according control agent (chondroitin sulfate, Ctrl) for 45 min. Then, samples were incubated for 3h with differentially pretreated Cy5-labeled FeO-DEX and assayed by FACS analysis (see figure S1). The graph denotes the relative MFI of Cy5 in liver NPC populations and is representative for 2 experiments.

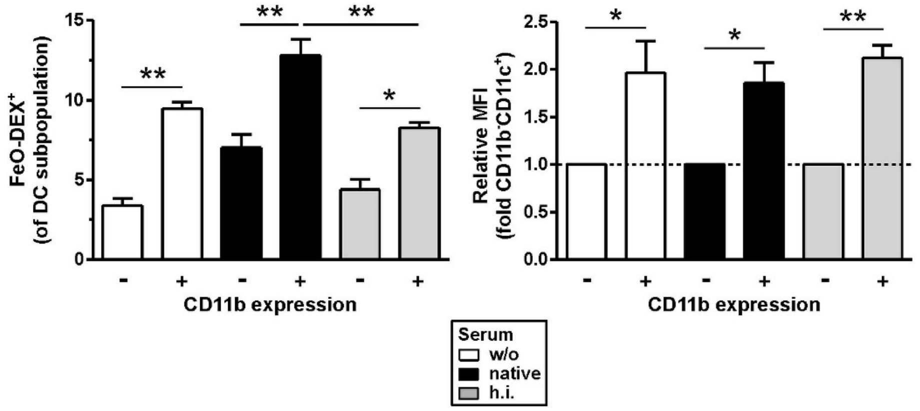

Figure S6. CD11b⁺ splenic DC bind FeO-DEX at higher extent. Spleen cell were incubated for 3h with differentially pretreated (left untreated [w/o], pretreated with native or heat-inactivated [h.i.] serum) FeO-DEX. The extent of FeO-DEX binding was assessed by FACS analysis (see figure S1). Data denote the frequencies of $Cy5^+$ cells (left panel) and the relative MFI of Cy5 (right panel) of CD11b-deficient (-) and CD11b-expressing (+) splenic DC (mean±SEM of 3 experiments). Statistical differences between groups are indicated (one way ANOVA, Tukey test). *p<0.05, **p<0.01.

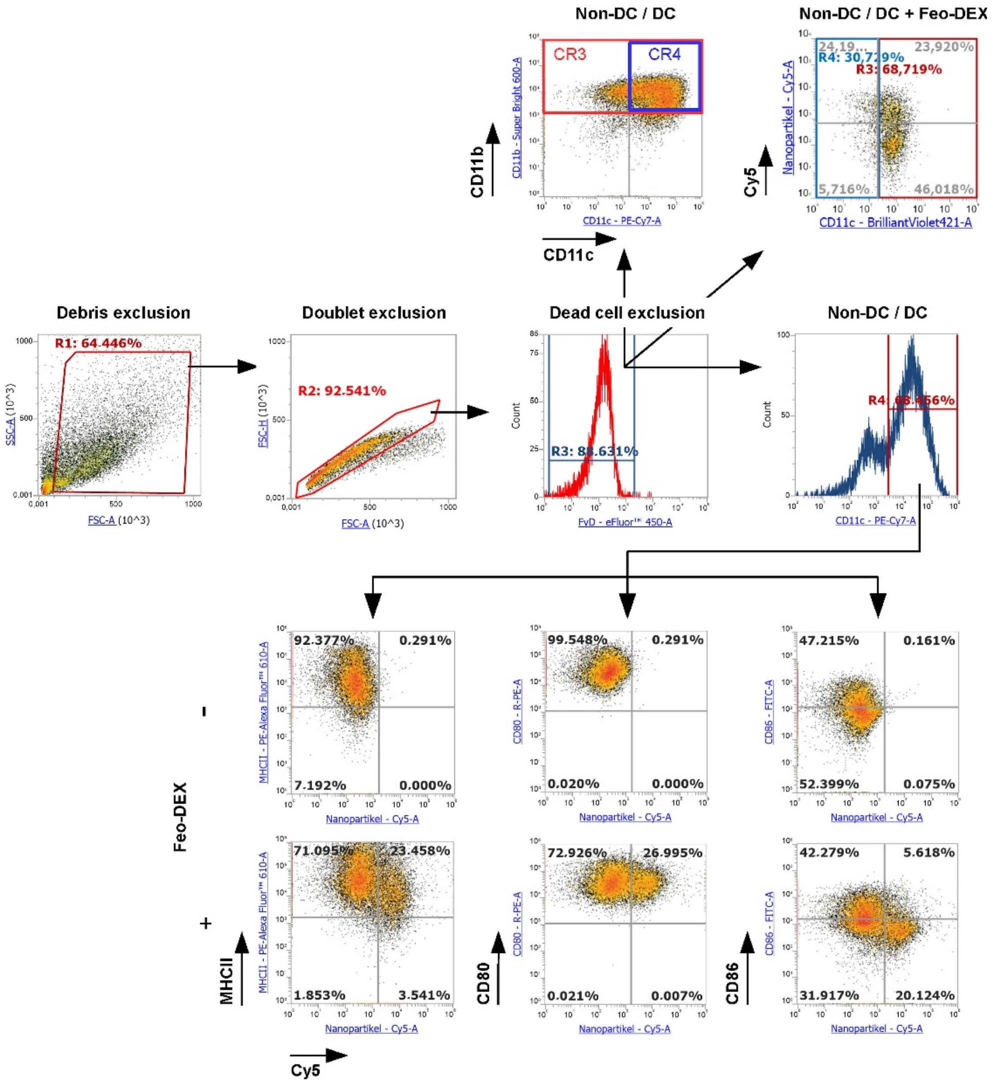

Figure S7. Gating strategy of BMDC. Middle panel: Debris, doublets and dead cells were excluded. Upper panel: Within the BMDC culture, non-DC and DC commonly express CD11b (CD11b/CD18, CR3), whereas only DC express CD11c (CD11c/CD18, CR4) (left plot). Both cell types engage carbohydrate-coated NC as shown for native serum-pretreated Cy5-labeled FeO-DEX (right plot). Lower panel: Assessment of DC activation markers (MHCII, CD80, CD86) in CD11c^{+} DC left untreated and after over-night incubation with native serum-pretreated FeO-DEX is shown.

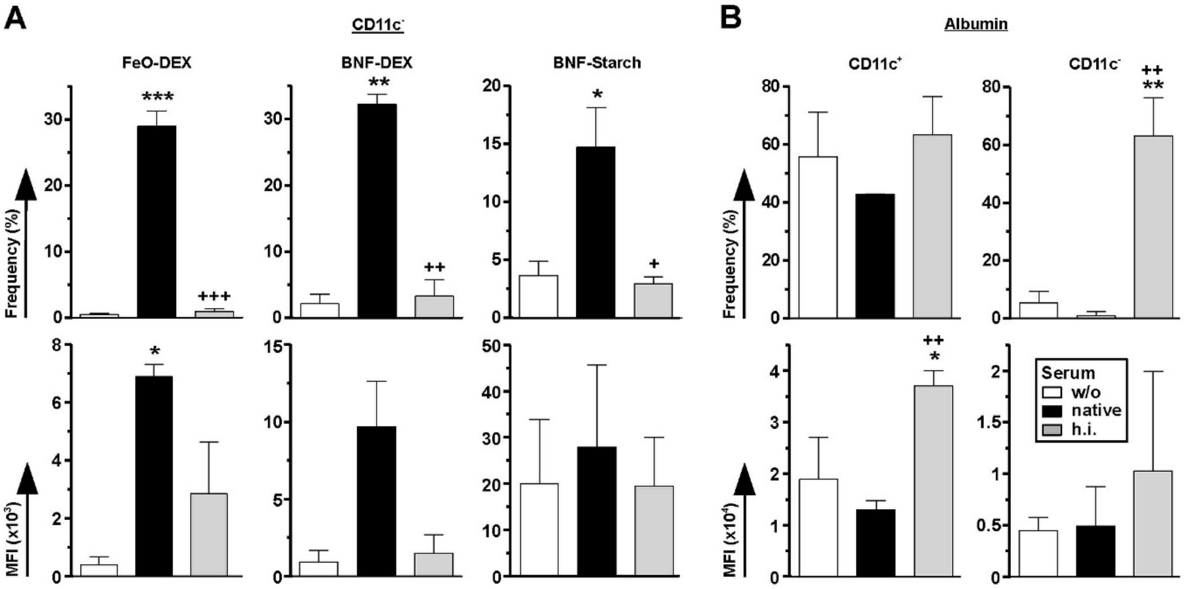

Figure S8. Macrophages (CD11b^{+}CD11c^{-}) within BMDC cultures engage complement-opsonized NC at higher extent. BMDC cultures were incubated for 3h with differentially pretreated (left untreated [w/o], pretreated with native or heat-inactivated [h.i.] serum) types of fluorescence-labeled (A) carbohydrate-coated and (B) albumin NC. The extent of NC binding was assessed by flow cytometry (see figure S7). Data denote the frequencies (upper panel) of NC-positive CD11c^{-} non-DC (A) and both DC and non-DC (B), and the relative MFI of NC-specific fluorescence (lower panel) of non-DC (A) and non-DC/DC (B) (mean±SEM of 3-4 independent experiments). Statistical differences versus *w/o serum and $^{+}$native serum are indicated (one way ANOVA, Tukey test). $^{*,+}$p<0.05, $^{**,++}$p<0.01, $^{***,+++}$p<0.001.

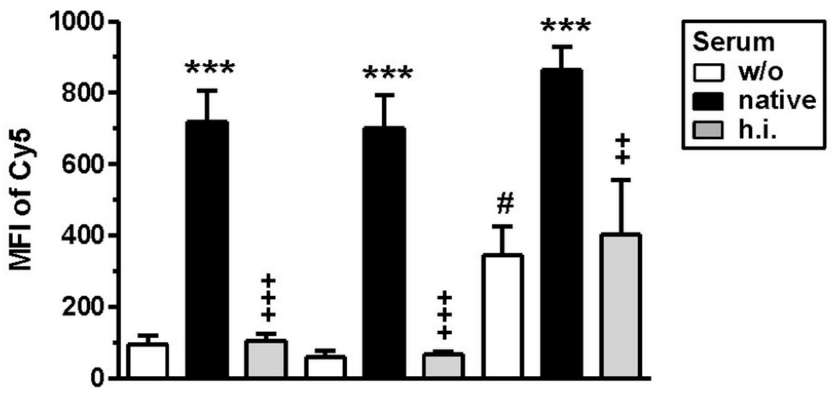

Figure S9. B-1 cells bind FeO-DEX at higher extent than B-2 cells. Spleen cells were incubated for 3h with differentially pretreated (left untreated [w/o], pretreated with native or heat-inactivated [h.i.]

serum) FeO-DEX. Data denote the Cy5 intensity (MFI) of total B cells ($CD19^+$), B-2 cells ($CD11b^-CD19^+$) and B-1 cells ($CD11b^+CD19^+$), given as the mean±SEM of 3 experiments. Statistical differences *versus w/o, +versus native serum (same B cell population), #B-1 versus B-2 (according group) are indicated (one way ANOVA, Tukey test). $^{\#}p<0.05$, $^{++}p<0.01$, $^{***,+++}p<0.001$.

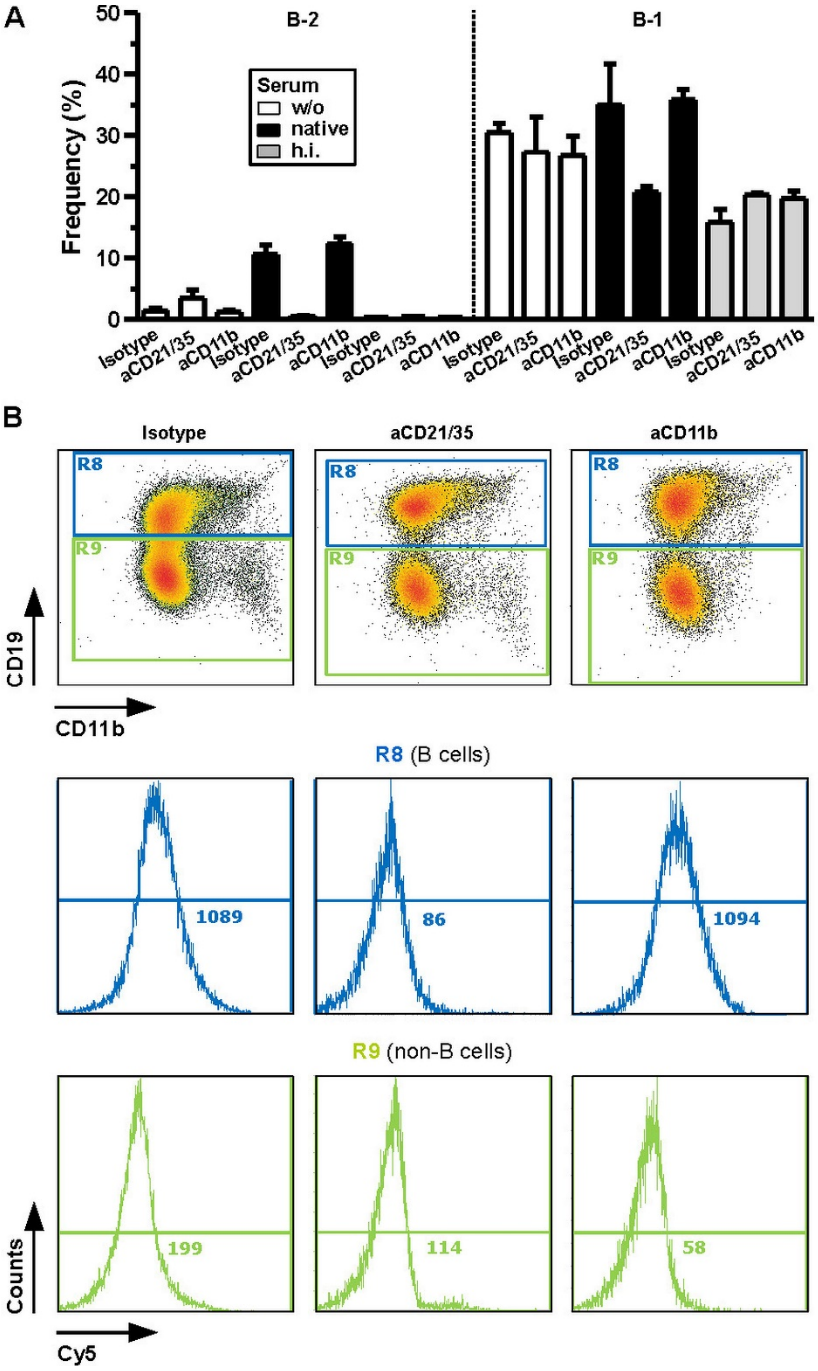

Figure S10. B cells bind complement-opsonized FeO-DEX via CR1/2. Spleen cells were pretreated in parallel with anti-CD21/CD35 (CR1/2) and anti-CD11b (CR3) blocking antibody, and an according isotype control antibody (each 5 µg/ml). Differentially pretreated FeO-DEX (left untreated [w/o], pretreated with native or heat-inactivated [h.i.] serum) were added after 45 min, and samples were incubated over-night. (**A**) The frequencies of $Cy5^+$ B-2 (left panel) and B-1 (right panel) cells (see figure

6) were assessed by FACS analysis (mean±SD of 2 experiments). (**B**) Histograms denote for CD19^{+} B cells (R8) and CD19^{-} non-B cells (R9) (upper panel) Cy5 intensities (MFI) (lower panel).
